# MicroRNAs-Based Inter-Domain Communication between the Host and Members of the Gut Microbiome

**DOI:** 10.3389/fmicb.2017.01896

**Published:** 2017-09-27

**Authors:** Maggie R. Williams, Robert D. Stedtfeld, James M. Tiedje, Syed A. Hashsham

**Affiliations:** ^1^Department of Civil and Environmental Engineering, Michigan State University, East Lansing, MI, United States; ^2^Center for Microbial Ecology, Michigan State University, East Lansing, MI, United States; ^3^Department of Plant, Soil, and Microbial Sciences, Michigan State University, East Lansing, MI, United States

**Keywords:** microRNAs, inter-domain communication, inter-kingdom communication, gut microbiome, homeostasis, environmental exposure, host-commensal

## Abstract

The gut microbiome is an important modulator of host gene expression, impacting important functions such as the innate immune response. Recent evidence suggests that the inter-domain communication between the gut microbiome and host may in part occur via microRNAs (small, non-coding RNA molecules) which are often differentially expressed in the presence of bacteria and can even be released and taken up by bacteria. The role of microRNAs in microbiome–host communication in intestinal diseases is not fully understood, particularly in diseases impacted by exposure to environmental toxicants. Here, we review the present knowledge in the areas of microbiome and microRNA expression-based communication, microbiome and intestinal disease relationships, and microRNA expression responses to intestinal diseases. We also examine potential links between host microRNA–microbiota communication and exposure to environmental toxicants by reviewing connections between (i) toxicants and microRNA expression, (ii) toxicants and gut diseases, and (iii) toxicants and the gut microbiome. Future multidisciplinary research in this area is needed to uncover these interactions with the potential to impact how gut-microbiome associated diseases [e.g., inflammatory bowel disease (IBD) and many others] are managed.

## Introduction

Inter-domain molecular communication plays a key role in host–gut microbiome interactions and symbiosis. MicroRNAs (small, non-coding RNAs that regulate gene expression post-transcriptionally) are emerging as a key mode for this communication (Zhou et al., 2017). It is hypothesized that pathogens modulate the expression of host microRNAs to enhance their survival. Regulation of host microRNAs impacts various biological pathways (e.g., innate immune response) through the regulation of host gene expression (Bartel, 2004).

**Figure 1:**
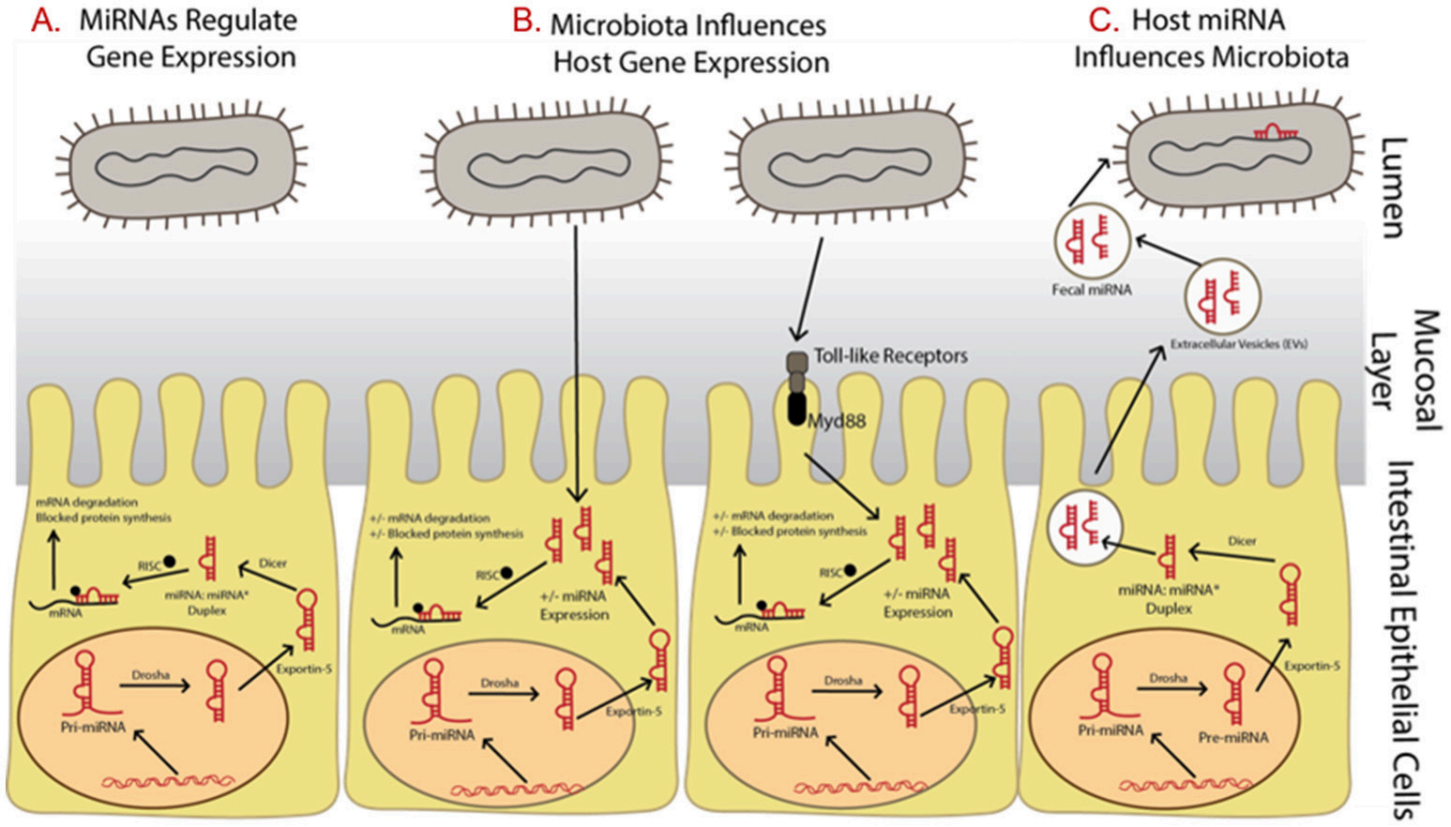
Summary of relationships between microRNAs and microbiota and the impact on gene expression regulation. **(A)** MicroRNAs begin as precursor hairpin loops, generated in the cell nucleus, exported to the cytosol, and processed by Dicer into two structures, the mature microRNA strand and a rapidly degraded passenger strand (often labeled with ^*^). **(B)** Microbiota have been shown to regulated microRNA expression, possibly through toll-like receptor/Myd88—dependent pathways. **(C)** The host may be influencing its gut microbiome by releasing fecal microRNAs, which are taken up by bacteria.

Dysbiosis of the commensal community has been linked with many gut-related diseases including irritable bowel syndrome (IBS), Crohn's disease, and gastric cancer (Round and Mazmanian, 2009), as well as many other disorders including those connected by the gut-brain axis such as autism, depression, and anxiety (Dinan et al., 2013) though many questions remain unanswered (McCarville et al., 2016; Aziz et al., 2017) and cause and effect has not been established (Degruttola et al., 2016). Three important interactions between the host and gut microbiome involving microRNAs are clear, however, including: (i) microRNAs regulate host gene expression (Figure 1A), (ii) gut microbiota influences host microRNA expression (Figure 1B), and (iii) the host influences the gut microbiota through the release of microRNAs (Figure 1C). It has also been suggested that the host itself may regulate its microbiota but evidence for this is still in its initial stages (Liu et al., 2016).

At the time of this review, over 2,500 microRNAs are known in humans (miRBase Registry; www.mirbase.org; Griffiths-Jones et al., 2008; Kozomara and Griffiths-Jones, 2014). Primary microRNAs are initially transcribed in the cell nucleus then cleaved by the enzyme Drosha into pre-microRNAs. They are then exported into the cytoplasm where they are processed by the enzyme Dicer. To regulate gene expression, microRNAs are assembled with Argonaute and GW182 into the RNA-induced silencing complex (RISC). They bind by partial complementarity of the last 7–8 bases to the 3′ untranslated region (3′ UTR) of messenger RNAs (mRNAs), thus blocking translation and preventing mRNA degradation (Figure 1A; Bartel, 2004). It is well-known that a single microRNA can target many mRNAs and a single mRNA can have many microRNAs that target it (Taganov et al., 2007). It has also been suggested that microRNAs control protein expression variability by decreasing variability for lower expressed proteins and increasing variability for those highly expressed (Schmiedel et al., 2015). This allows microRNAs to have many regulatory roles in various cellular processes and many microRNAs are thus implicated in various diseases. In fact, forced overexpression of microRNAs has led to tumorigenesis in laboratory studies (He et al., 2005). In addition, over 100 microRNAs are shown to circulate in serum and their use as potential biomarkers of disease has been suggested (Chen et al., 2008). MicroRNA levels in serum and plasma have also been reported as up/down regulated between cancer patient samples and healthy controls, depending on the cancer type and microRNA studied (e.g., reviewed in Peng and Croce, 2016).

Regulation of host gene expression is one means of communication between the gut microbiota and host through the manipulation of host microRNA expression (Figure 1B). In fact, microRNA expression profiles are shown to be very different when comparing gut samples collected from traditional or colonized mice with germ free mice (Dalmasso et al., 2011; Singh et al., 2011; Xue et al., 2011). These differentially expressed microRNAs can in turn affect gene expression regulation of a number of gut related diseases. At present, the exact mechanism by which microbiota influence microRNA expression is unclear, though it has been suggested it may involve toll-like receptors and Myd88-dependent pathways (Xue et al., 2011; Larsson et al., 2012). However, the host may be influencing its gut microbiome through the release of microRNA in extracellular vesicles which are taken up by microbes and may affect microbial growth (Figure 1C; Liu et al., 2016).

Finally, the effects of outside influences on both the gut microbiome and microRNA expression are important. For example, environmental toxicants have been shown to be associated with differential microRNA expression and disease. MicroRNAs have been proposed as biomarkers of environmental exposure as they are frequently differentially expressed following an exposure event. The gut microbiome is also often directly impacted by exposure to synthetic chemicals due to direct metabolism of chemicals, chemicals altering enzymatic activity, or the induction of dysbiosis (Claus et al., 2016). Dysbiosis could further impact disease, though there is no evidence to date except in the case of antibiotics (Becker et al., 2016).

## Gut microbes influence host microRNA expression

The influence of pathogenic microorganisms (such as *Listeria monocytogenes, Salmonella enterica*, and *Helicobacter pylori*) on host microRNA expression has been extensively reviewed (e.g., Maudet et al., 2014). Far fewer studies exist reporting the influence of commensal bacteria on microRNA expression (Table 1; reviewed in Masotti, 2012; Runtsch et al., 2014) though specific gut commensals are not evaluated. Most studies focusing on the gut microbiome and host microRNAs have used mixed microbial communities as part of traditional mice and compared them to germ-free or colonized mice. These have focused on ileum, colon, and caecum because host microRNA expression is expected to be tissue-specific. Measurement of microRNA levels was carried out mostly by qPCR (Singh et al., 2011; Archambaud et al., 2013; Dai et al., 2015) but in certain studies microarray-based relative expression was obtained (Dalmasso et al., 2011; Xue et al., 2011). A total four studies measured larger mice microRNA panels for higher throughput screening (Dalmasso et al., 2011; Singh et al., 2011; Xue et al., 2011; Archambaud et al., 2013) while others measured only targeted microRNAs (Xue et al., 2011; Dai et al., 2015). One study that also included human ulcerative colitis (UC) patients, was interested in measuring a single microRNA (miR-193a-3p) because of its relevance to UC from earlier studies (Dai et al., 2015). As seen in Table 1, the number of differentially expressed microRNAs in response to commensal gut bacteria was between 5 and 16 in the three studies that measured the whole mouse panel of microRNAs. The changes in expression level are significant but seldom drastically different (e.g., in Singh et al., 2011 the maximum fold change for downregulated microRNAs was 0.2 and for upregulated microRNAs was 4.6).

**Table 1:** Studies relating mixed microbial communities from traditional or colonized mice to host microRNA expression.

**Results**					
**Comparison**	**Sex; Weight; Age of individuals**	**Sample type**	**Differentially expressed microRNAs** (↑ Upregulation, ↓ Downregulation)	**Gene or functional targets of differentially expressed microRNAs** (↑ Upregulation, ↓ Downregulation)	**References**
Germ-free (*n* = 6) vs. colonized Swiss Webster mice (*n* = 6)	Female; N/A; 8 weeks	Ileum, Colon	Ileum: miR-298↑	Abcc3↓ (directly targeted by miR-665 3' UTR and validated with *in vitro* studies)	Dalmasso et al., 2011
			Colon: miR-128↑, miR-200c^*^↑, miR-342-5p↑, miR-465c-5p↓, miR-466d-3p↓, miR-466d-5p↓, miR-665↓, miR-683↓		
Germ-free (*n* = 5) vs. traditional Swiss Webster mice (*n* = 5)	Male; N/A; 5 weeks	Caecum	miR-21^*^↓, rno-miR-351↓, miR-351↓, miR-487b↓, miR-467a↓, miR-27b^*^↓, miR-148a↓, miR-145↑, miR-183↑, miR-133a↑, miR-150↑, miR-672↑, miR-181a^*^↑, rno-miR-664↑, miR-455↑, miR-138^*^↑, let-7g^*^↑	54 genes related to intestinal barrier function (potential targets determined computationally)	Singh et al., 2011
Not infected (*n* = 3) vs. infected conventional C57BL/6 mice with *Listeria monocytogenes* (*n* = 3)	Female; N/A; 9-12 weeks	Ileum	Conventional: miR-143↓, miR-148a↓, miR-200b↓, miR-200c↓, miR-378↓	Protein encoding genes (potential targets determined computationally)	Archambaud et al., 2013
Not infected (*n* = 3) vs. infected germ-free C57BL/6 mice with *Listeria monocytogenes* (*n* = 3)			Germ-free: miR-194↓, miR-378↑		
Germ-free (*n* = 3) vs. traditional C57BL/6 mice (*n* = 3)	Female; N/A; 8-10 weeks	Dendritic cells	miR-10a↓	Il-12/IL-23p40↑ (directly targeted by miR-10a 3' UTR and validated with *in vitro* studies)	Xue et al., 2011
Traditional (*n* = 3) vs. germ-free Swiss Webster mice (*n* = 3)	Male; N/A; 10-12 weeks	Aorta	miR-204↓	Sirt1↑ (directly targeted by miR-204 3' UTR and validated with *in vitro* studies)	Vikram et al., 2016
Healthy (*n* = 7) vs. colitis- induced C57BL/6 mice (*n* = 7) Healthy (*n* = 12) vs. active ulcerative colitis patients (*n* = 11)	Mice: Female; 18-22 g; 8 weeks Humans: N/A	Colon	miR-193a-3p↓	PepT1↑ (validated with *in vitro* studies) Colonic inflammation↑	Dai et al., 2015

One of the earliest reports of the impact of the gut microbiome on microRNA expression used germ-free mice colonized with gut microbiota from specific pathogen-free (SPF) mice (Dalmasso et al., 2011). Briefly, Swiss Webster SPF mice (8 weeks; female) were colonized then introduced to germ-free mice cages. After 4 days, all mice were sacrificed and colons and ileum were collected. MicroRNA expression profiles were determined via microarray and quantitative reverse transcription polymerase chain reaction (qRT-PCR) by comparing tissues from germ-free (*n* = 6) and colonized mice (*n* = 6). Increased expression of miR-128, miR-200c^*^, and miR-342-5p and decreased expression of miR-465c-5p, miR-466d-5p, miR-665, and miR-683 was observed in colon tissues (Table 1). Increased expression of miR-298 was observed in ileum tissues. Using *in vitro* studies, it was confirmed that miR-665 targets the Abcc3 gene (ATP-binding cassette transporter) 3′ UTR which was downregulated in colonized mice.

Caecal microRNA signatures have also been compared between germ-free (*n* = 5) and conventional (*n* = 5) Swiss Webster mice (male; 5 weeks; Singh et al., 2011). Overall, 334 microRNAs were detected in both groups with 16 differentially expressed between them (including miR-21^*^, rno-miR-351, miR-351 which were downregulated). Computational approaches and gene expression analysis revealed the potential targets of each microRNA which were involved in regulating intestinal barrier genes and immune system regulation, though these genes were not validated *in vitro* as targets of the microRNAs.

MicroRNA expression profiles from both traditional and germ-free C57BL/6 mice have also been shown to be influenced following oral *L. monocytogenes* infection (Archambaud et al., 2013). Briefly, 9–12 week old female mice were divided into four groups (*n* = 3 per group) including (i) germ-free, not infected, (ii) germ-free, infected, (iii) conventional, not infected, and (iv) conventional, infected. After infection with *L. monocytogenes*, ileum samples were collected and microRNA expression profiles were analyzed. Two microRNAs (miR-194 and miR-378) were differentially expressed between infected and non-infected germ-free mice while five microRNAs (miR-143, miR-194, miR-200b, miR-200c, and miR-378) were differentially expressed between infected and non-infected conventional mice. This suggests that the conventional mice were responding more to the *Listeria* infection than their germ-free counterparts. Finally, it was observed that the ten most highly expressed microRNAs in this study could be considered as “signature” microRNAs that are always present in high abundances.

Some studies have measured selected microRNAs focusing on one or two based on their role in specific functions. Xue et al. studied expression of miR-10a because of its role in regulating the innate immune response through targeting IL-12/IL-23p40 (Xue et al., 2011). It was reported that miR-10a expression in intestinal dendritic cells of conventional C57BL/6 mice was significantly lower compared to germ-free mice (female; 8–10 weeks old; *n* = 3 per group). The microbiota was shown to regulate miR-10a using TLR-TLR ligand interactions and a MyD88-dependent pathway. Furthermore, the IL-12/ IL-23p40 was increased and the 3′ UTR was determined to be a direct target of miR-10a (validated with *in vitro* studies). UC mice with high IL-12/IL-23p40 expression also had lower expression of miR-10a in intestinal tissues compared with healthy mice, suggesting the importance of miR-10a in disease, though this may not be due to direct affects.

The gut microbiome may also be regulating host microRNAs and function in regions beyond the gut. In a recent study, the decreased expression of miR-204 was observed in the aorta of germ-free (*n* = 3) as compared to traditional Swiss Webster mice (*n* = 3; Vikram et al., 2016). All mice in the experiment were males, 8–10 weeks in age. Following decreased expression of miR-204, its target, Sirt1 (sirtuin1 lysine deacetylase) was significantly increased. In fact, the microbiome was shown to remotely govern miR-204. After administration of broad-spectrum antibiotics in mice to control the microbiome, Sirt1 expression was decreased which resulted in impaired endothelial function, specifically endothelium-dependent vasorelaxation.

Host genetics have been reported to shape the gut microbiome community structure which in turn influences host metabolism (Goodrich et al., 2014) but a recent report suggests that the host may also influence its gut microbiome through fecal extracellular microRNAs (Liu et al., 2016). It was observed that intestinal epithelial cells (IECs) are major sources of extracellular fecal microRNAs, due to their ability to secrete exosome-like vesicles. Fecal microRNAs are protected from degradation due to protection by (i) entrapment in extracellular vesicles and (ii) association to high-density lipoproteins or argonaute complexes (Creemers et al., 2012). Interestingly, overall abundance of fecal microRNAs was increased in germ-free mice vs. SPF colonized mice. Furthermore, in IEC-microRNA-deficient mice (mice without the enzyme Dicer), bacterial growth in the gut was uncontrolled but after undergoing fecal microRNA transplantation, homeostasis was observed, suggesting that control of bacterial growth may be due to the extracellular fecal microRNAs. However, microRNAs are involved in many important gene regulation processes (not just uptake into bacterial cells as the authors point out) which may impact dysbiosis via more indirect means. It is also noted that knocking out Dicer may not completely eliminate microRNAs processed in the cell, though they are significantly reduced.

These studies although limited in number indicate that microRNAs serve as an important communication channel between the gut microbiome and the host. Differential microRNA expression in turn regulates the host gene expression, potentially impacting pathways and host physiology and disease status. Unfortunately, a number of confounding factors exist as most of the experiment designs are distinctly different. For example, the influence of the use of male or female mice can impact expression profiles as well as age, different tissue types and different number of replicates. Table 1 shows that all studies discussed in this section used different age animals and were split in their use of males or females. Influence of specific members of the gut microbiome on these expression profiles could also affect results. Future studies are needed to determine the context of these results in overall health.

## MicroRNAs associated with select gut diseases

Differential expression of microRNAs as it pertains to diseases has been studied extensively for gut-associated diseases such as inflammatory bowel disease (IBD; Kalla et al., 2015), Crohn's disease (Kalla et al., 2015), UC (Kalla et al., 2015), and gastric cancer (Ishiguro et al., 2014). In fact, these microRNAs are both tissue-specific (Kalla et al., 2015) and circulating (Paraskevi et al., 2012) microRNAs are differentially expressed in patients with IBD compared to healthy controls. Circulating and other cell-free microRNAs could also serve as useful disease biomarkers (Zahm et al., 2011), as they only require a small blood or fecal sample employing relatively simple sampling procedures. It has also been suggested that circulating microRNAs may travel the bloodstream and regulate gene expression in distant cells (Creemers et al., 2012).

As such, extracellular microRNAs have been shown to be differentially expressed in various diseases as compared with healthy controls. For example, miR-29a collected from blood microvesicles, small bowel tissues, and colon tissues has been shown to be significantly increased in patients with IBS (Zhou et al., 2010). MiR-29a modulates an increase in intestinal barrier permeability due to its complementarity to the 3′ UTR of the glutamine synthetase gene, thereby blocking production of glutamine synthetase, an enzyme that was reported in the study to decrease intestinal barrier permeability (Zhou et al., 2010). Therefore, increased levels of miR-29a could lead to an increase in permeability which was observed during cell culture experiments. Specifically, IBS patients with increased intestinal barrier permeability showed increased expression of miR-29a compared with IBS with normal permeability. Similarly, expression levels of miR-150 and miR-342-3p in peripheral blood have also been shown to increase in patients with IBD, with a fold-change greater than 1.6 (Fourie et al., 2014). Though regulatory mechanisms were predicted using an Integrated Pathway Analysis (IPA) network, it was not experimentally investigated as part of the study. It was suggested that because miR-150 is interacts with protein kinase AKT2, it may thus affect inflammatory pathways associated with IBD. In addition, miR-342-3p may be important for pain signaling, motility in the colon, and smooth muscle function.

MicroRNAs have also been shown to be involved in the complications typically associated with Crohn's disease. For example, the miR-200 family are involved in fibrogenesis in the intestine (Chen et al., 2012). Using *in vitro* studies with IECs, fibrogenesis was induced using transforming growth factor β1 (TGFβ1) which induces fibrosis in Crohn's disease. It was shown that TGFβ1 not only induced fibrosis but also inhibited the expression of miR-200b. Administration of miR-200b *in vitro* also protected IECs, in part, from fibrosis and suggests this could be due to miR-200b inhibiting zinc finger E-box-binding homeobox 1 and 2 (ZEB1 and ZEB2). Furthermore, to determine if miR-200b could be used as a potential biomarker of fibrosis in Crohn's disease, serum of patients with Crohn's disease and fibrosis (*n* = 10) were compared to serum from healthy controls (*n* = 16) and Crohn's disease patients without fibrosis (*n* = 10). MiR-200b was shown to be significantly upregulated in all comparisons for patients with Crohn's disease and fibrosis, suggesting it could be used as a potential biomarker.

Select studies report that microRNAs could be useful biomarkers for the detection of cancers (e.g., Calin and Croce, 2006), due to significant differences in expression levels observed from a variety of samples between patients and healthy controls. Ohshima et al. have suggested that gastric cancer cells may use hsa-let-7a to promote oncogenesis (Ohshima et al., 2010). Results show that let-7a, which targets oncogenes (RAS, HMGA2) and suppresses the development of cancer, is released by gastric cancer cells (such as AZ-P7a cells) into their exosomes, thus maintaining oncogenesis. Indeed, it was found that let-7 family was abundant in both intracellular and extracellular environments, while the low metastatic cell line AZ-521 had much lower levels in both environments, which could result in increased expression levels in gastric cancer.

Characterizing the altered microRNA expression in certain gut diseases is important for understanding their role in disease as well as in the development of treatment options. Though the relationship between microRNA expression and gut diseases has been extensively studied, the full extent of this relationship and the importance of the gut microbiota on this is less clear.

## Microbes associated with gut diseases

Dysbiosis of gut microbial communities that results in the loss of host-microbiota symbiosis often results in a shift from symbiont to pathobiont. This shift in microbial community structure is important in the development, incidence, recurrence, and treatment of major gut diseases such as IBD. Though many pathogenic organisms are also involved in these diseases (e.g., *H. pylori* infection increases the risk of gastric cancer; Wroblewski et al., 2010), this section focuses on commensals or pathobionts that are not always pathogenic. The importance of commensals in maintaining overall gut homeostasis is clear, as evidenced by the success of fecal transplantation in the treatment of gut diseases such as UC (Borody et al., 2003).

One well-characterized change is the differences in the phylum *Firmicutes* in IBD. *Faecalibacterium prausnitzii* is an important gut commensal as it is a major producer of butyrate, which plays a key role in gut physiology and modulation of the immune system (Wrzosek et al., 2013). Specifically, reduced abundance of *F. prausnitzii* has been observed in Crohn's disease patients (*n* = 22) as compared to healthy controls (*n* = 27; Sokol et al., 2008). Other reports have suggested increased abundances of *F. prausnitzii* in pediatric Crohn's disease patients (*n* = 13) as compared to healthy controls (*n* = 12; Hansen et al., 2012). This increased abundance was also correlated with overall reduced bacterial diversity, something that has been observed in many other studies for Crohn's disease, IBD, and UC (Ott et al., 2004; Lepage et al., 2011; Hansen et al., 2012). Interestingly, the decrease in abundance of *F. prausnitzii* in UC and Crohn's disease has also associated with an increase in other gut commensals such as *Bifidobacterium* and *Lactobacillus* (Wang et al., 2014).

Another predominant commensal of the gut microbiome is *Bacteroides* including *Bacteroides fragilis*, which expresses polysaccharide A (PSA), a compound that modulates the host immune response by inducing Treg and cytokine expression (Troy and Kasper, 2010). PSA in itself can provide protection against colitis by repressing pro-inflammatory cytokines (Mazmanian et al., 2005). In an elegant review by Zhou et al., based on an extensive analysis of the literature from 1990 to 2016, it was determined that abundances of *Bacteroides* spp. in Crohn's disease and UC patients were significantly lower than in healthy controls (Zhou and Zhi, 2016). Unfortunately, though typically commensal, some strains of *B. fragilis* produce enterotoxins which can cause illness and diarrhea. Due to its ability to induce cytokine expression and widespread abundance, enterotoxigenic *B. fragilis* can result in persistent inflammation and induction of colitis and colonic tumors, as validated in multiple intestinal neoplasia mice (Wu et al., 2009). This occurs through a signal transducer and activator of transcription-3 (Stat3) and T helper type 17 (Th17)-dependent pathway, as Wu et al. noted.

Only one study has connected the differential expression of microRNAs in response to the microbial community observed in gut diseases (Dai et al., 2015). Specifically, in C57BL/6 mice (18-22 g; 8-week old; female; *n* = 7) colitis was induced providing mice with filtered sterile water containing 3% dextran sodium sulfate for 8 days while control mice (*n* = 7) were given untreated water. In colitis- induced mice, miR-193a-3p was downregulated while colonic PepT1 (di/tripeptide transporter) and overall colonic inflammation was upregulated (Table 1). In fact, PepT1 uptakes bacterial products suggesting a direct relationship between microRNAs and microbiota and increased expression of PepT1 is associated with IBD and indeed treatment with antibiotics resulted in reduced inflammation. Furthermore, after treatment with miR-193a-3p mimics, inflammation was also reduced. Similar expression profile results were also observed in human subjects when comparing healthy (*n* = 12) and active UC patients (*n* = 11).

Dysbiosis of the gut microbiome is also linked with gut diseases such as Crohn's disease and UC. Through evaluating specific community shifts (such as *F. prausnitzii* and *Bacteroides*), probiotic-based treatments targeting that dysbiosis could be used to revert to homeostasis, though much more research is required in this area as cause and effect has not been evaluated and conflicting reports exist (Degruttola et al., 2016; McCarville et al., 2016; Aziz et al., 2017). Though the relationship of microRNA expression to gut diseases as well as the relationship of gut microbiome dysbiosis to gut diseases has been studied, their investigation together (namely the communication that may be occurring therein) has not. For example, *F. prausnitzii* is decreased in Crohn's disease (Hansen et al., 2012) but this may be affecting the microRNA- based communication in some way. As both microRNAs and gut microbes are important in the development of certain gut diseases, it is evident that role of microRNAs cannot be overlooked in studies focusing on gut microbiome associated diseases.

## Links between exposure to environmental contaminants, microRNA expression, and the gut microbiome

Exposure to environmental contaminants can increase the risk for many diseases. A number of these contaminants alter genetics through DNA sequence mutation, DNA methylation, histone modifications, and differential microRNA expression in the host (Hou et al., 2012). In fact, host microRNAs have been shown to be biomarkers of environmental exposure to various chemical agents (Vrijens et al., 2015) as they are differentially expressed following exposure to contaminants (Hou et al., 2012). Examples of environmental contaminants that may alter host microRNA expression include cigarette smoke, aluminum, arsenic, bisphenol A, diethyl phthalate (DEP), formaldehyde, polycyclic aromatic hydrocarbons (PAH), hexahydro-1,3,5-trinitro-1,3,5,-triazine (RDX) and 2,3,7,8-tetrachlorodibenzo-p-dioxin (TCDD) (as reviewed in Vrijens et al., 2015).

Some of these reports have associated environmental exposure with differential microRNA expression and gut diseases. For example, TCDD and other AhR activators are associated with colitis (Benson and Shepherd, 2011) and colorectal cancers (Xie and Raufman, 2015), BPA is implicated in colorectal cancer (Chen et al., 2015), and PAH may lead to digestive tract cancers (Diggs et al., 2011). Though most of the research involving differential microRNA expression in response to contaminants involves other tissues such as liver (Zhang and Pan, 2009; Szabo and Bala, 2013), some effects related to the gastrointestinal tract (particularly those related to gastric and colorectal cancer) are also reported. For example, Mullany et al. observed an association between host differential microRNA expression, cigarette smoke, and rectal or colorectal cancer (Mullany et al., 2016). In this study, 306 host microRNAs were differentially expressed in smokers, with 200 directly associated and 41 inversely associated with tumor phenotypes for either colon or rectal cancer. These findings strongly suggest that the exposure to cigarette smoking may impact cancer development through differential microRNA expression. Smoking has been associated with other gut related diseases (Mahid et al., 2006) though conflicting reports exist (Rosenfeld and Bressler, 2012).

**Figure 2:**
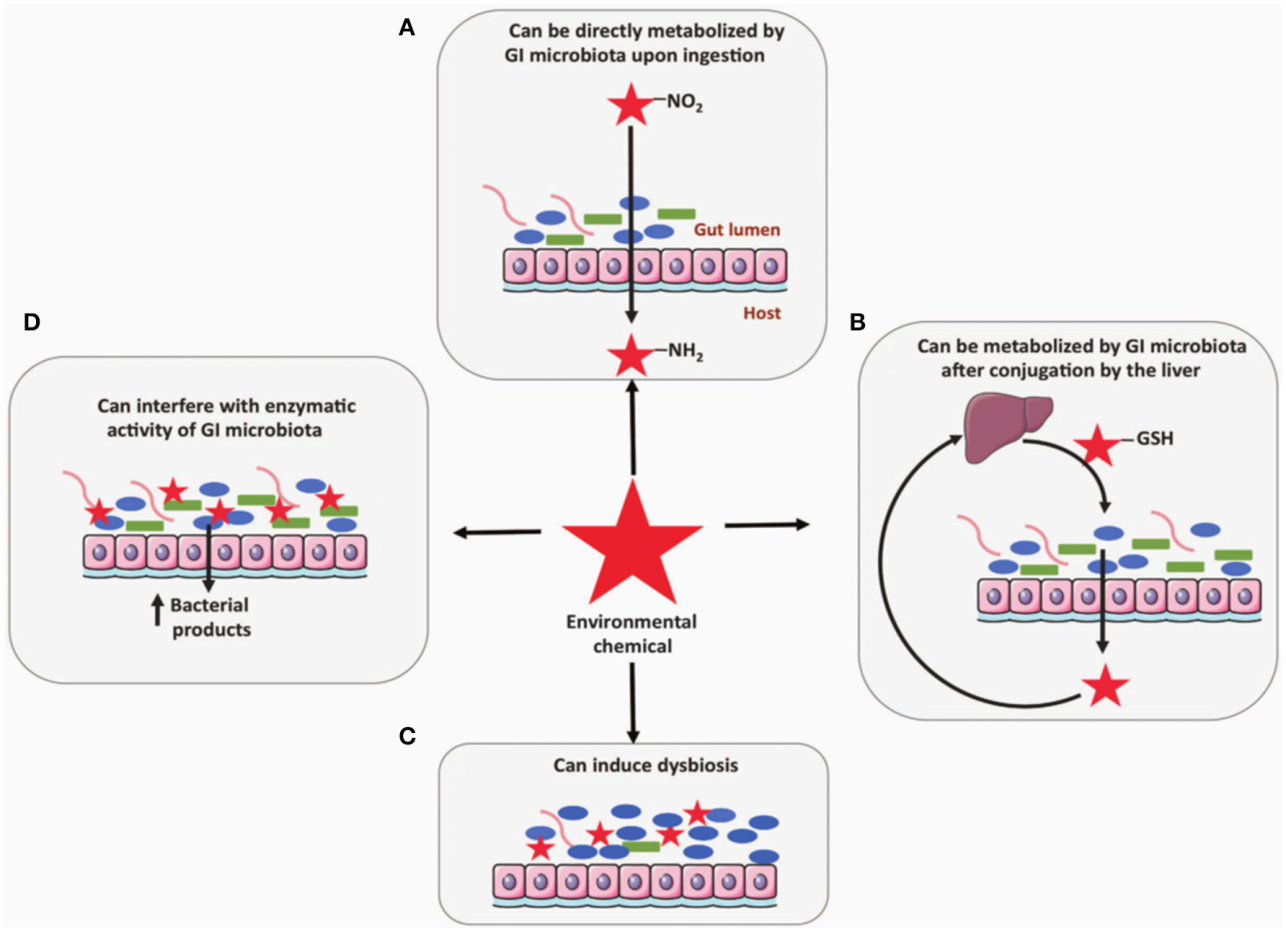
Four mechanisms the gut microbiome may be influenced by exposure to environmental contaminants include: **(A)** direct metabolism, **(B)** metabolism following conjugation in the liver, **(C)** induction of dysbiosis, and **(D)** interfering with enzymatic activity (Claus et al., 2016). Reproduced from Claus et al. (2016) with permission under the Creative Commons License by Nature Publishing Group.

It has been suggested that the gut microbiome may be impacted by exposure to environmental contaminants by four possible mechanisms: (i) direct metabolism, (ii) metabolism following conjugation in the liver, (iii) interfering with enzymatic activity, and (iv) induction of dysbiosis (Claus et al., 2016; Figure 2). Certain chemicals are directly metabolized by gut microbiota including PAH, Nitrotoluene, DDT, PCB, and pesticides (Claus et al., 2016). Some of these, such as nitrated PAH, can form conjugates after metabolism by microbiota that are carcinogenic and more hazardous to the host than the initial chemical (Möller, 1994). Exposure to environmental contaminants has also been associated with dysbiosis of the gut microbiome. For example, smokers with active Crohn's disease were found to have significantly higher levels of *Bacteroides* than healthy controls (Benjamin et al., 2012). Specifically, healthy controls who also smoked had higher levels of *Bacteroides-Prevotella* than non-smokers, while Crohn's disease patients who smoked had higher *Bifidobacteria* and *Bacteroides-Prevotella* and lower *F. prausnitzii*. Unfortunately, research related to connecting dysbiosis of the gut microbiome with gut diseases is still in its initial stages. Further efforts are needed to define the impact of these linkages on disease.

## Challenges ahead and concluding remarks

Overall, the research reviewed here suggests that microRNAs may play a crucial role in communications between the gut microbiome and the host to maintain gut homeostasis and prevent disease. In this review, we have discussed potential interactions between the gut microbiome and host microRNAs, microRNAs and gut diseases, and gut diseases and the gut microbiome. These complex relationships suggest that perhaps the symbiotic relationship we share with the gut microbiome is indeed co-evolved, down to the nucleic acid level. Furthermore, as recent research highlights the host regulation of the microbiome through fecal microRNAs (Liu et al., 2016), the microbiome may not be simply regulating host homeostasis, but the relationship between the host and the microbiome works together to maintain symbiosis. The question of “who is controlling whom?” is an interesting one, though the answer remains unclear and highlights that maybe the host and its bacteria are continually controlling each other to maintain ideal circumstances for both.

Despite these relationships, many aspects of the gut microbiome-host interactions remain unknown. First, the relationship and communication between the gut microbiome and microRNAs as they relate to gut diseases has not been fully evaluated. There is also the need to define the link between gut diseases and dysbiosis. Second, many outside factors (such as environmental exposure to toxicants) impact the gut microbiome, differential microRNA expression, and gut diseases. Unfortunately, studies that investigate the combined effect of all of these factors together do not yet exist. These may prove to be important in microRNA-based communication with the gut microbiome, particularly since they have all been shown to be connected separately. Until all aspects are researched together, cause and effect cannot be defined. Future studies investigating the impact of toxicants on human health would also benefit from evaluating the outside variables such as the gut microbiome and differential microRNA response. Interdisciplinary studies that include the fields of toxicology, microbiology, and human health in particular would help bridge the gaps in current knowledge related to microRNA-based communication with the gut microbiome.

## Author contributions

Manuscript preparation, including literature review, figure drawing, and editing was completed by MW. Manuscript concept development and editing was completed by RS, JT, and SH.

### Conflict of interest statement

The authors declare that the research was conducted in the absence of any commercial or financial relationships that could be construed as a potential conflict of interest.
